# Supplementary material for: Lysyl Oxidase-like Protein Recognizes Viral Envelope Proteins and Bacterial Polysaccharides against Pathogen Infection via Induction of Expression of Antimicrobial Peptides
Source: Viruses. 2022 Sep 18;14(9):2072. doi: 10.3390/v14092072 (PMC9500624; doi:10.3390/v14092072)
Supplement: Supplementary file 1 [file viruses-14-02072-s001.zip › viruses-1899974-supplementary.pdf]

Supplementary information

Table S1. Sequences of the primers used in this research.

Primers	Sequence (5′-3′)
RT-PCR and qRT-PCR	
Actin-RT-F	AGTAGCCGCCCTGGTTGTAGAC
Actin-RT-R	TTCTCCATGTCGTCCCAGT
Dorsal-RT-F	GCAATGCTGGTAACCTGGCTA
Dorsal-RT-R	CTATGGGATTTTGGTCAATACACTTT
Ef1-α-RT-F	GGATTGCCACACCGCTCACA
Ef1-α-RT-R	CACAGCCACCGTTTGCTTCAT
ie1-RT-F	GACTCTACAAATCTCTTTGCCA
ie1-RT-R	CTACCTTTGCACCAATTGCTAG
LOXL-RT-F	CTGAGACCTCCCTTTACGA
LOXL-RT-R	CAGAGCCACAAACTCCATC
Relish-RT-F	AGGATGAAGATGAGGAGGAA
Relish-RT-R	GAGATGTCAATGCCCGAGT
STAT-RT-F	CGCCCAAATGGAATGAT
STAT-RT-R	CGGATAAAGGTAAAGGAGGTA
VP28-RT-F	AGCTCCAACACCTCCTCCTTCA
VP28-RT-R	TTACTCGGTCTCAGTGCCAGA
Alf-A1-F	CTGGTCGGTTTCCTGGTGGC
Alf-A1-R	CCAACCTGGGCACCACATACTG
Alf-B1-F	CGGTGGTGGCCCTGGTGGCACTCTTCG
Alf-B1-R	GACTGGCTGCGTGTGCTGGCTTCCCCTC
Alf-C1-F	CGCTTCAAGGGTCGGATGTG
Alf-C1-R	CGAGCCTCTTCCTCCGTGATG
Alf-C2-F	TCCTGGTGGTGGCAGTGGCT
Alf-C2-R	TGCGGGTCTCGGCTTCTCCT
Alf-E1-F	TCCTAACCACGCAGTGCTTTGCTAATG
Alf-E1-R	GCTTTTCGGATTTGCCTTCGATGTTTG
CrusI-1-F	TGCTCAGAACTCCCTCCACC
CrusI-1-R	TTGAATCAGCCCATCGTCG
CrusI-3-F	CTCCACCACTCTCGCACTAACA
CrusI-3-R	TGATGGTCTCAGATTGGGGC
q16S-891-F	TGGAGCATGTGGTTTAATTCGA
q16S-1003R	TGCGGGACTTAACCCAACA
Recombinant expression	
LOXL-ORF-F	TACTCAGGATCCGACGAGGAGGGGAAGATCCGG
LOXL-ORF-R	TACTCAGAATTCAGGCCGGGTGAGAGAACAATT
LOXL-SRCR1-F	TACTCAGGATCCATCCGGCTCGTCGACGGCCGC
LOXL-SRCR1-R	TACTCAGAATTCATCACACACGACCCCCGCCGA
LOXL-SRCR2-F	TACTCAGGATCCGTGAGACTCGTCGGAGGCCGG
LOXL-SRCR2-R	TACTCAGAATTCATACAGGTGACGCCCCCGAT
LOXL-lysyl oxidase-F	TACTCAGGATCCTGTATCGATAAAATAGCTGAT
LOXL-lysyl oxidase-R	TACTCAGAATTCAGGCCGGGTGAGAGAACAATT
RNA interference	
LOXL-Ri-F	GCGTAATACGACTCACTATAGGGTGACGGTTGGAGCATTCTGG

LOXL-Ri-R	GCGTAATACGACTCACTATAGGAAGCATCGGTTCCTTGGTTGA
Gfp-Ri-F	GCGTAATACGACTCACTATAGGTGGTCCCAATTCTCGTGGAAC
Gfp-Ri-R	GCGTAATACGACTCACTATAGGCTTGAAGTTGACCTTGATGCC
Dorsal-Ri-F	GCGTAATACGACCACTATAGGCCATAGAGCTAGATA
Dorsal-Ri-R	GCGTAATACGACTCACTATAGGTCAGTACCCAAGTGT
Relish-Ri-F	GCGTAATACGACTCACTATAGGTGCTGGTTGAGAAAGTTAGG
Relish-Ri-R	GCGTAATACGACTCACTATAGGATGTCAGGTGTTGGCATAGA
STAT-Ri-F	GCGTAATACGACTCACTATAGGCATGGCTCTGGCAGATAAG
STAT-Ri-F	GCGTAATACGACTCACTATAGGGATGATTGAGACTCGCACC

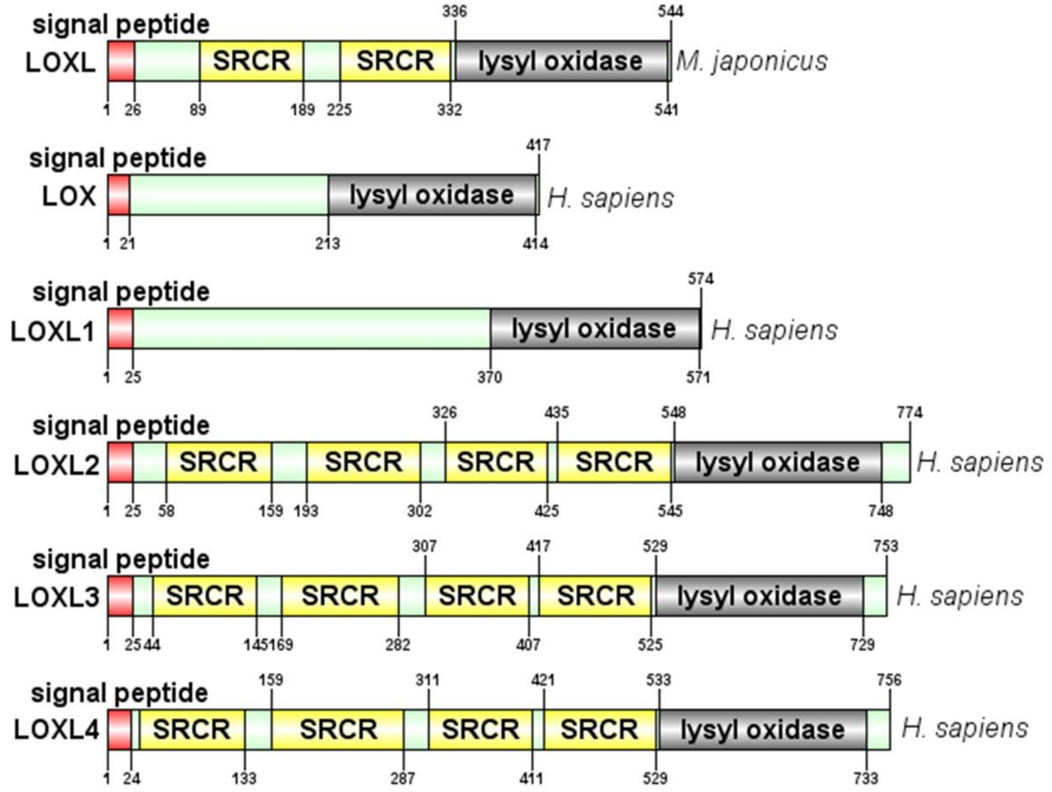

Figure S1. Comparison of domain architectures of the LOX/LOXLs in *M. japonicus* and *H. sapiens*. The LOX/LOXL amino acid sequences from *H. sapiens* were obtained from GenBank (LOX, GenBank accession number: AAB23549.1; LOXL1, AAA50162.1; LOXL2, AAH00594.1; LOXL3, AAK63205.1; LOXL4, AAL27543.1).

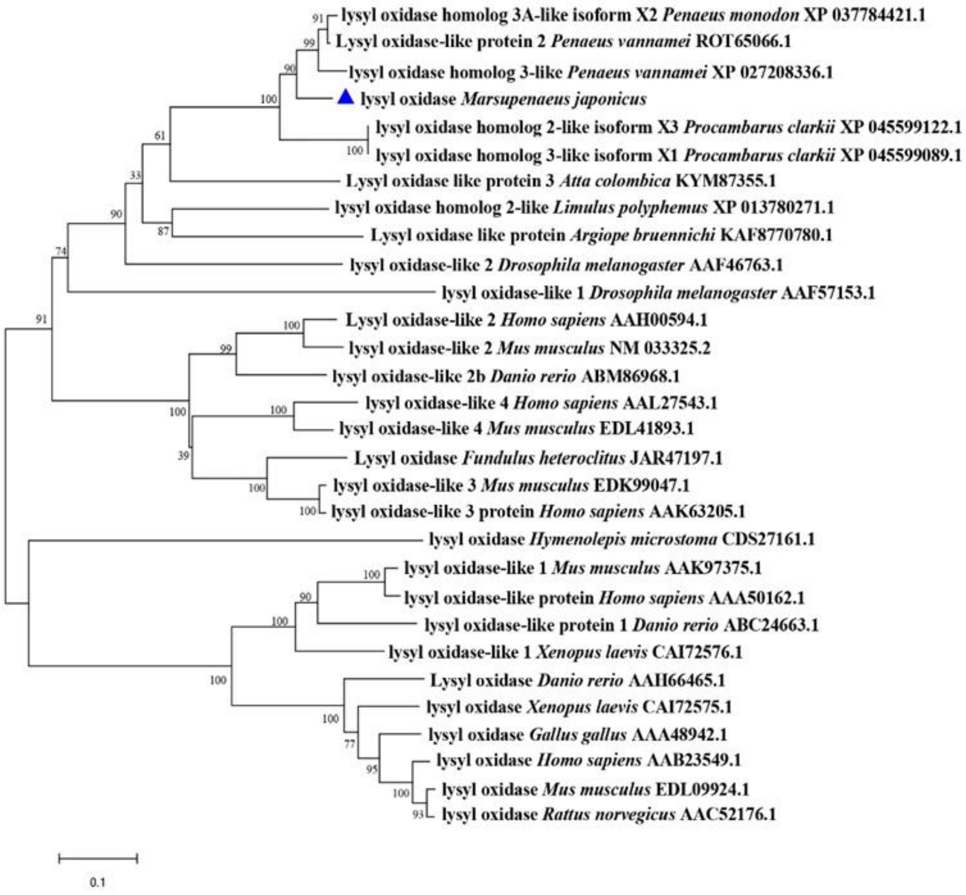

Figure S2. Phylogenetic tree of LOX/LOXLs from different species. The LOX/LOXL sequences of different species were obtained from GenBank, and the NJ tree was established using MEGA 7.0. The results were repeated 1000 times via bootstrapping. LOXL of *M. japonicus* is marked with a blue triangle.

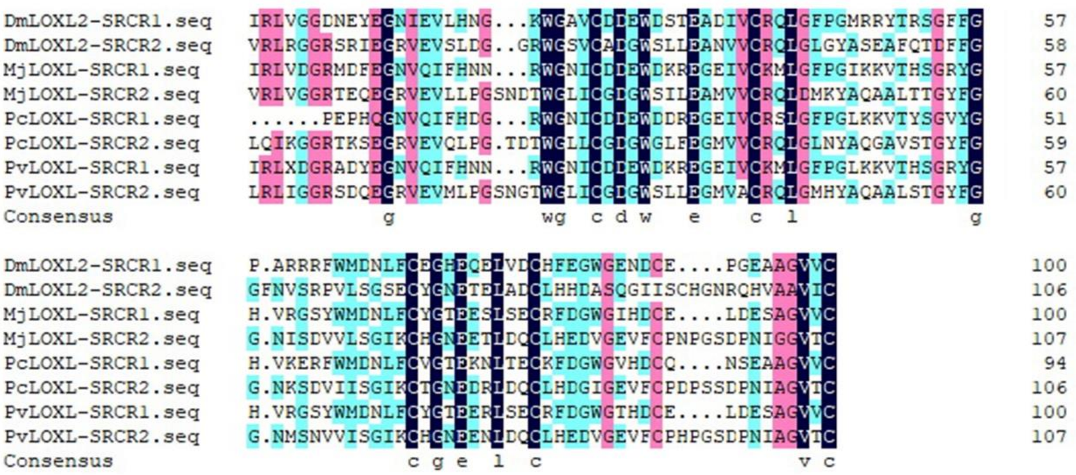

Figure S3. Sequence alignment of SRCR1 and SRCR2 domains in different shrimp species. The SRCR1 and SRCR2 sequences of different species were obtained from GenBank, and the alignment was established using DNAMAN.

.

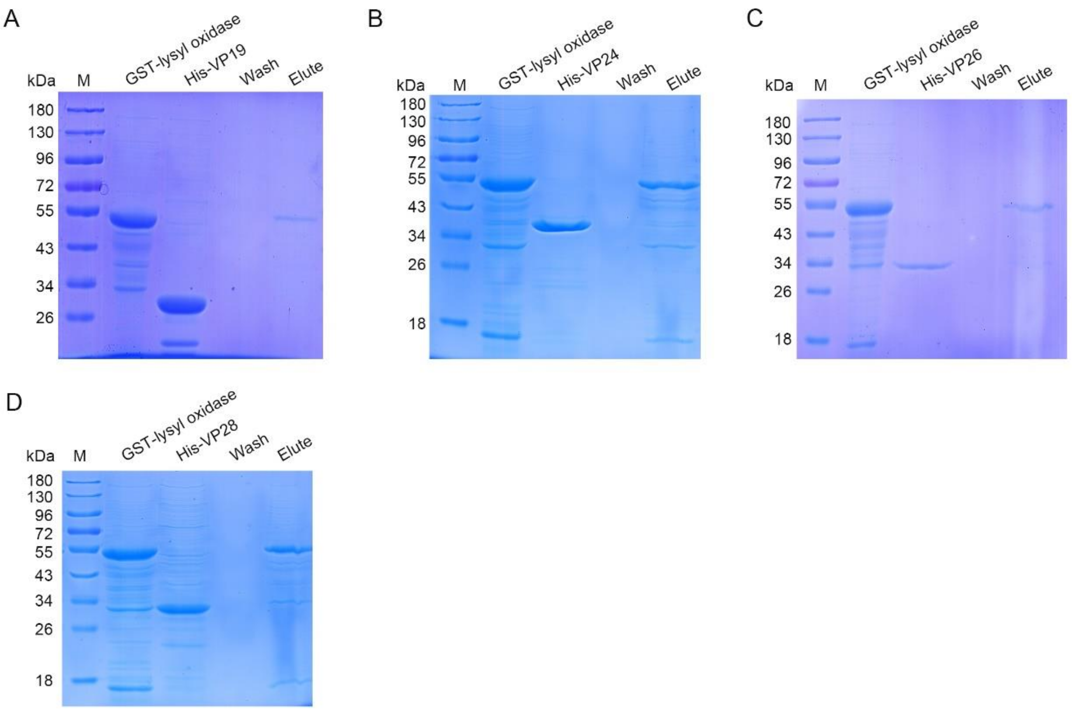

Figure S5. Lysyl oxidase domain did not interact with envelope proteins of WSSV. (A, B, C, D) GST-pulldown assays to detect the interaction of lysyl oxidase domain with recombinant VP19, rVP24, rVP26, and rVP28. Lysyl oxidase domain could not bind to envelope proteins

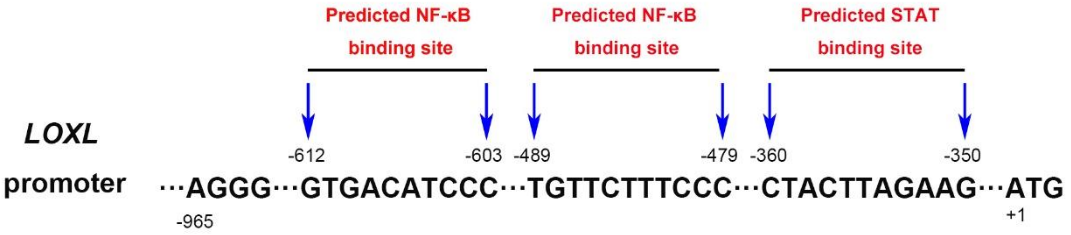

Figure S5. Prediction of LOXL promoter region binding to NF-κB or STAT in *M. japonicus*. The promoter sequence of *LoxL* was obtained online from genome of *M. japonicus* (https://blast.ncbi.nlm.nih.gov/Blast.cgi). The predicted promoter binding sites were obtained from the online analysis website, JASPAR (https://jaspar.genereg.net/).
